# Differences in Anthropometric Parameters of Children in Six European Countries

**DOI:** 10.3390/children10060983

**Published:** 2023-05-31

**Authors:** Csilla Semánová, Gergő J. Szőllősi, István Ilyés, Greet Cardon, Julie Latomme, Violeta Iotova, Yuliya Bazdarska, Jaana Lindström, Katja Wikström, Sandra Herrmann, Peter Schwarz, Eva Karaglani, Yannis Manios, Konsantinos Makrilakis, Luis Moreno, Esther M. González-Gil, Imre Rurik, The Feel4Diabetes-Study Group

**Affiliations:** 1 Department of Family and Occupational Medicine, Faculty of Medicine, University of Debrecen, 4032 Debrecen, Hungary; ilyesi@yahoo.com; 2 Doctoral School of Health Sciences, University of Debrecen, 4032 Debrecen, Hungary; szollosi.gergo@etk.unideb.hu (G.J.S.); rurik.imre@med.unideb.hu (I.R.); 3 Department of Health Informatics, Faculty of Health Sciences, University of Debrecen, 4032 Debrecen, Hungary; 4 Department of Movement and Sports Sciences, Ghent University, B-9000 Ghent, Belgium; greet.cardon@ugent.be (G.C.); julie.latomme@ugent.be (J.L.); 5 Department of Social Medicine and Health Care Organization, Medical University of Varna, 9002 Varna, Bulgaria; iotova_v@yahoo.com (V.I.); yuliya.bazdarska@gmail.com (Y.B.); 6 Department of Paediatrics, Medical University of Varna, 9002 Varna, Bulgaria; 7 Department of Public Health and Welfare, Finnish Institute for Health and Welfare, FI-00271 Helsinki, Finland; jaana.lindstrom@thl.fi (J.L.); katja.wikstrom@thl.fi (K.W.); 8 Department for Prevention and Care of Diabetes, Faculty of Medicine Carl Gustav Carus, Technische Universität Dresden, 01062 Dresden, Germany; sandra.herrmann@uniklinikum-dresden.de (S.H.); peter.schwarz@uniklinikum-dresden.de (P.S.); 9 Paul Langerhans Institute, Dresden of Helmholtz Zentrum München, University Hospital and Faculty of Medicine, 01307 Dresden, Germany; 10 German Center for Diabetes Research, 40225 Neuherberg, Germany; 11 Department of Nutrition and Dietetics, School of Health Science and Education, Harokopio University, 17671 Athens, Greece; ekaragl@hua.gr (E.K.); manios@hua.gr (Y.M.); 12 First Department of Propaedeutic Medicine, Athens Medical School, National and Kapodistrian University, Laiko General Hospital, 11527 Athens, Greece; kmakrila@med.uoa.gr; 13 Growth, Exercise, Nutrition and Development (GENUD), Research Group, University of Zaragoza, 50009 Zaragoza, Spain; lmoreno@unizar.es (L.M.); esthergg@unizar.es (E.M.G.-G.); 14 IBER, Fisiopatología de la Obesidad y Nutrición, Instituto de Salud Carlos III, 28222 Madrid, Spain; 15 Hungarian Society of Nutrition, 4002 Budapest, Hungary; 16 Department of Family Medicine, Semmelweis University, 1085 Budapest, Hungary

**Keywords:** body weight, body height, BMI of children, child growth, Belgium, Bulgaria, Finland, Greece, Hungary, Spain, Feel4Diabetes

## Abstract

Background: The databases of children’s anthropometric parameters are often outdated, rarely representative and are not always available at an international level. Objectives: To present children’s anthropometric parameters in six European countries that contributed to the Feel4Diabetes project and find country-specific differences. Design/Setting: The Feel4Diabetes study was performed between 2016 and 2018, targeting children in Belgium, Bulgaria, Finland, Greece, Hungary and Spain. The current study presents data from the baseline and the yearly follow-up anthropometric measurements. Subjects: In total, 20,832 measurements of children (48.7% boys) between 6 and 10 years of age were conducted. Main outcome measure: weight, height, BMI. Results: Belgian boys had the lowest body weight and height, while Greek boys had the highest body weight, and Finnish had the highest body height. The highest proportion of overweight (percentile above 85%) and obese boys (percentile above 95%) was in Greece, followed by Hungarian, Spanish, Bulgarian and Finnish boys. In contrast, Belgian boys had the lowest ratio in both categories. Among girls, Greece had the highest; Belgium had the lowest body weight; Finland was the highest in all age categories. The ratio in the overweight range was the highest in Greece, followed by Spanish, Bulgarian and Hungarian girls, who were second in the obese category. Finnish girls had lower and Belgian girls had the lowest ratio in both BMI categories. All the detailed data are presented in tables, and the trends are figures. Conclusions: Our study presents fresh and comparable anthropometric data of children between 6 and 10 years of age in six European countries, supporting the need for appropriate obesity prevention.

## 1. Background

Childhood obesity represents one of the most important and challenging public health problems in developed and even less developed countries. Its metabolic and health-related consequences in this age period and later in adulthood are well known [1,2]. The home environment is important, particularly for young children, and can be improved by educating and empowering families, supported by school- and community-based intervention [3,4].

Anthropometric measurements are part of regular children’s care and are usually performed by pediatricians, midwives or other staff members of school health services. Registered data could help providers determine if a child is growing properly and can indicate when the child’s health and well-being are at risk. To evaluate the individual, registered data can serve as a comparison to (national) databases, which are generated from national(wide) surveys and measurements of the same cohorts regarding gender and age. These data are usually presented in tables or in age-growth charts.

Two of the most important health indicators for children that are regularly measured include their individual growth patterns (weight and height) together with BMI (Body Mass Index). It is a simple weight-for-height index that provides the most useful population-level measure of overweight and obesity worldwide, as it is the same for both sexes and for all ages, including children [5,6,7].

According to a professional consensus, children two years of age or older with a BMI between the 85th and 94th percentile on age-growth charts are considered overweight, while children with a BMI higher than the 95th percentile are considered obese [8,9,10]. The BMI z-score is a quantitative measure of the deviation of a specific BMI percentile from the mean of that population. A positive z-score indicates that a child is heavier, while in the case of a negative z-score, a child is lighter than the mean. Thus, a z-score can compare the BMI of a given child to the BMI distribution for a population of children of the same age and sex [4,11].

Available national databases of the child populations are often outdated or do not always provide appropriate representatives. In recent decades there was a shift in the anthropometric parameters of children, which was caused by the “acceleration” of their growth, which closely depended on their changes in living conditions, improved nutrition and other socio-economic circumstances. One of the most used databases was published 20 years ago, based on data from six different countries [12].

The *Feel4Diabetes Project* was a large school- and community-based study that aimed to promote a healthy lifestyle, including healthy eating and increased physical activity, in order to alleviate obesity and obesity-related metabolic risk factors in families at risk for Type-2 Diabetes (T2D) in Europe. The project ran between 2014 and 2019, and the actual program implementation had a total duration of two years (2016–2018) [13]. The *Feel4Diabetes Project*, founded by the European Commission, offered an excellent option to register anthropometric parameters: firstly, to measure a high number of children and, secondly, to make a comparison between populations measured in the six participating countries (Belgium, Bulgaria, Greece, Finland, Hungary and Spain) [14,15].

## 2. Aim

The current study aimed to present data of the anthropometric parameters of children in the six participating countries over a period of two years and compare national trends.

## 3. Method

### 3.1. Study Population

*Feel4Diabetes* was a European community-based study that included an intervention component at the family, school and community levels [16]. Recruitment was based on a standardized, multi-stage sampling procedure and was conducted within selected provinces in the participating countries, targeting vulnerable population groups at high risk of developing T2D. In *Low- and Middle-Income Countries (LMICs)*, including Bulgaria *(BG)* and Hungary *(HU)*, all municipalities within the participating regions were eligible for recruitment, while in *High-Income Countries* (*HICs*) such as Belgium *(BE)*, Finland *(FI)*, families within low socioeconomic status *(SES)* municipalities were recruited. In Greece *(GR)* and Spain *(ES),* low SES municipalities were defined as those with the lowest educational level and/or the highest unemployment rates, as retrieved from official resources and local authorities within each country [15]. Measurements were taken at the baseline of the project, such as the start of the academic year, in the first three grades of obligatory (primary level) education, while intervention and follow-up occurred in the upcoming 2 years. The same method and selection procedures were used in every participating country as has been already described [14,15].

For this study, 20.832 children were included (48.7% boys), and their distribution for age, gender and country are detailed in the tables.

### 3.2. Measurements

The anthropometric indices of children were measured by trained researchers using standardized protocols and equipment that were calibrated before the start of the measurements (in each time period). Participants were asked to remove heavy clothing and shoes and to stand still in an erect position during the measurements. Portable equipment was used (digital scales for weight, telescopic stadiometers for height, and non-elastic waist tape for waist circumference) [14,16]. Children were first measured at the start of the project when they were 6, 7 or 8 years old. The measurements were repeated every year, and the data of each child were considered at their respective year of age. (i.e., a child entering into the project at 6 years of age was allotted to the group of 6-year-old children. One year later, he/she already belonged to the group of 7 years old children, together with those who started at 7 years of age. (In the text, the exact age groups are abbreviated, i.e., 6–6.99 as 6 year). After calculating the BMI, the 85th percentile was the cut-off for overweight, while above the 95th percentile was considered obesity.

### 3.3. Statical Analysis

Descriptive statistics were performed on the merged data of 20,832 measurements in the period between 2016 and 2018, focusing on children aged 6–9.99 years. Proportions and 95% confidence intervals were calculated. These cases were considered significant, where the 95% confidence intervals did not overlap. Comparisons were not always made between all groups and categories.

### 3.4. Ethical Permission

*Feel4Diabetes-study* adhered to the Declaration of Helsinki and the conventions of the Council of Europe on human rights and biomedicine. Prior to initiating the intervention, all participating countries obtained ethical clearance from the relevant national ethical committees and local authorities (*in Hungary, by the National Committee for Scientific Research in Medicine; the approval number is 20095-1/2016/EKU (469/15)*). All parents/caregivers provided a signed consent form before being enrolled in the study [14,15].

## 4. Results

The data and their comparisons are presented in 2 tables and 8 figures.

**BOYS. Weight.** At the age of 6 y, Hungarian boys had the highest body weight, but from 7 to 9 y of age, Greek boys had the highest body weight. Belgian boys had a significantly lower body weight in each cohort, as presented in Table 1.

**Height.** At the age of 6 y, Belgian boys had the lowest *body height*. In the age group of 8 y, the Hungarian boys had the smallest height than other countries; this was significant compared to the data of Greek and Finnish boys. In the age group 9 y, Finnish boys were the tallest and significantly higher than Hungarians.

**BMI.** In the age group 6 y, Bulgarian boys had the lowest value, followed by the Belgian, without significant differences. In the older categories (7–9 y), Belgian boys always had the lowest BMI; however, boys in Greece had the highest.

**Table 1:** Anthropometric data of **boys** in each age group of 6 European countries. (BE = Belgium; BG = Bulgaria; FI = Finland; GR = Greece; HU = Hungary, ES = Spain).

Prevalence					
BOYS	Age-Group [Year]	*n*	Weight [kg]	Height [cm]	BMI [kg/m^2^]
**BE**	**6–6.99**	**139**	23.67 [23.04–24.29]	121.83 [120.90–122.76]	15.87 [15.62–16.13]
	**7–7.99**	**378**	26.00 [25.56–26.44]	127.32 [126.76–127.88]	15.97 [15.79–16.15]
	**8–8.99**	**610**	29.15 [28.73–29.57]	133.09 [132.62–133.56]	16.38 [16.22–16.54]
	**9–9.99**	**578**	32.12 [31.63–32.61]	138.04 [137.54–138.54]	16.77 [16.59–16.96]
**BG**	**6–6.99**	**37**	24.51 [23.24–25.78]	124.35 [123.01–125.68]	15.79 [15.15–16.43]
	**7–7.99**	**331**	28.18 [27.55–28.81]	128.39 [127.79–129.00]	16.99 [16.70–17.28]
	**8–8.99**	**593**	31.33 [30.80–31.86]	133.49 [133.01–133.96]	17.48 [17.25–17.71]
	**9–9.99**	**673**	34.93 [34.34–35.52]	138.20 [137.73–138.68]	18.16 [17.92–18.41]
**FI**	**6–6.99**	**13**	25.73 [23.84–27.62]	124.30 [121.80–126.81]	16.60 [15.74–17.47]
	**7–7.99**	**179**	27.66 [27.01–28.30]	128.38 [127.66–129.10]	16.72 [16.43–17.01]
	**8–8.99**	**414**	30.91 [30.34–31.48]	133.69 [133.13–134.25]	17.20 [16.97–17.44]
	**9–9.99**	**605**	34.70 [34.12–35.27]	139.25 [138.75–139.74]	17.76 [17.54–17.98]
**GR**	**6–6.99**	**237**	25.34 [24.73–25.95]	122.33 [121.68–122.98]	16.84 [16.54–17.14]
	**7–7.99**	**544**	29.25 [28.73–29.77]	128.11 [127.65–128.57]	17.69 [17.45–17.92]
	**8–8.99**	**875**	33.04 [32.56–33.52]	133.57 [133.19–133.94]	18.38 [18.18–18.59]
	**9–9.99**	**719**	36.77 [36.17–37.38]	138.49 [138.05–138.93]	19.02 [18.78–19.25]
**HU**	**6–6.99**	**34**	26.24 [23.99–28.48]	124.32 [121.94–126.71]	16.74 [15.82–17.66]
	**7–7.99**	**242**	27.72 [26.86–28.58]	127.35 [126.54–128.16]	16.93 [16.54–17.32]
	**8–8.99**	**490**	31.18 [30.49–31.87]	132.47 [131.87–133.07]	17.60 [17.31–17.90]
	**9–9.99**	**705**	34.91 [34.24–35.58]	137.79 [137.26–138.32]	18.21 [17.94–18.47]
**ES**	**6–6.99**	**164**	24.74 [24.08–25.40]	121.85 [121.07–122.63]	16.54 [16.25–16.84]
	**7–7.99**	**383**	27.63 [27.09–28.18]	127.37 [126.83–127.91]	16.92 [16.68–17.17]
	**8–8.99**	**626**	30.98 [30.47–31.48]	133.01 [132.56–133.47]	17.39 [17.18–17.61]
	**9–9.99**	**580**	34.03 [33.42–34.63]	137.69 [137.19–138.20]	17.82 [17.58–18.06]

The means of weight-, height- and BMI values, 95%CI of boys separately in 4 age categories during the project period.

**GIRLS. Weight.** Greek girls had the highest weight; the Belgian girls had the lowest body weight, which was significantly lower than their Hungarian counterparts. This was found for all age cohorts (see Table 2).

**Height.** The Finnish girls were the highest in all age categories. Compared to the Hungarians, at 6 years of age, the Belgian, Greek and Spanish girls were significantly smaller, while at 7 y, the Finnish and, at 8 y, the Bulgarians were significantly taller.

**BMI.** In the age group of 6 y, there was no significant difference between the countries. In the later age groups, Greek girls always had the highest values, though this was only significant at 9 y. Greek girls had the significantly highest BMI values. In age groups from 7 to 9 y, Belgian girls had the lowest BMI values compared to others.

**Figure 1:**
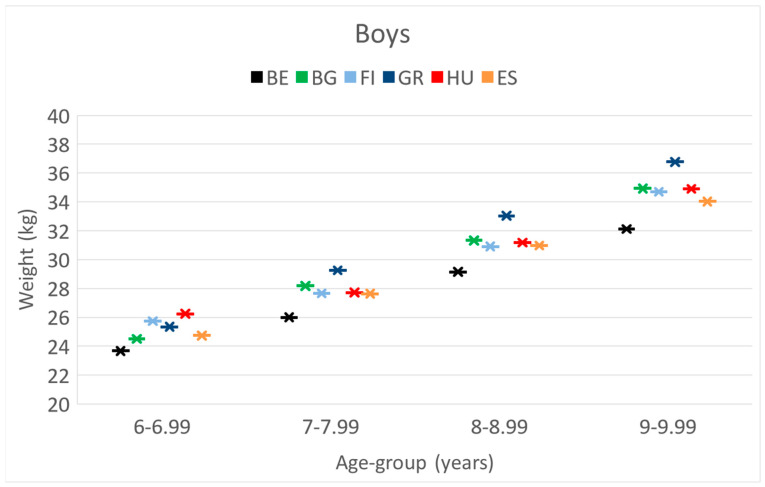
The increase in the mean values of weight [kg] and boys by their age [years] in the 6 countries. (BE = Belgium; BG = Bulgaria; FI = Finland; GR = Greece; HU = Hungary; ES = Spain).

**Figure 2:**
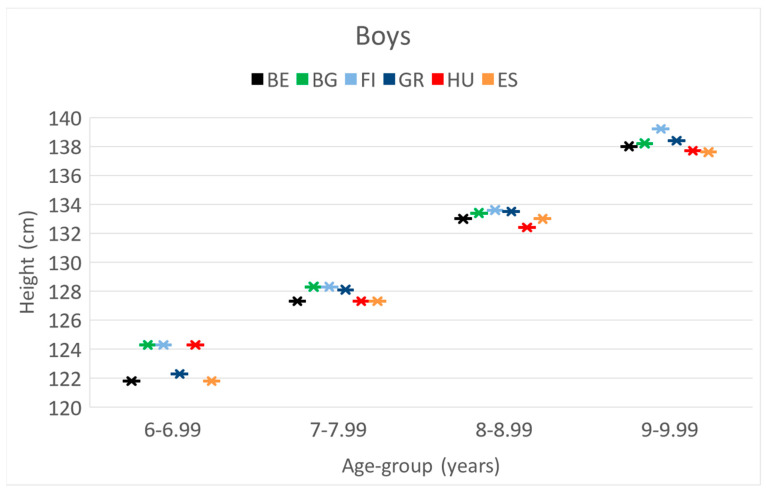
The increase in the mean values of the height [cm] of boys by their age [years] in the 6 countries. (BE = Belgium; BG = Bulgaria; FI = Finland; GR = Greece; HU = Hungary; ES = Spain).

**Figure 3:**
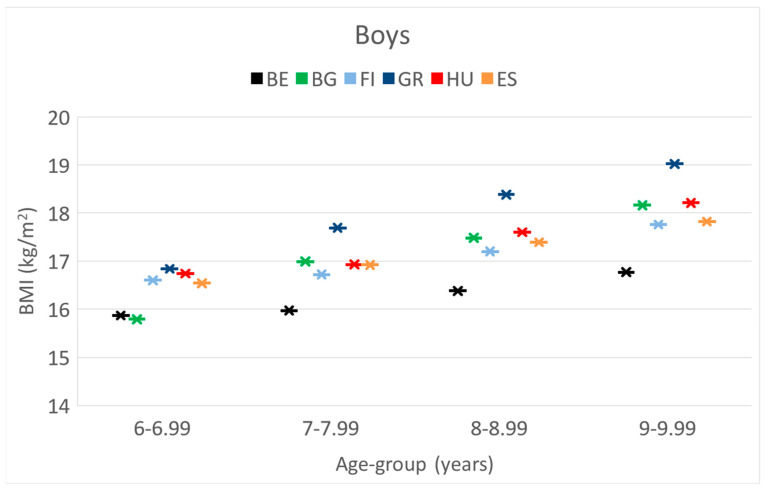
The increase in the mean values of BMI [kg/m^2^] of boys by their age [years] in the 6 countries. (BE = Belgium; BG = Bulgaria; FI = Finland; GR = Greece; HU = Hungary; ES = Spain).

These differences and trends are also presented in Figure 1, Figure 2 and Figure 3.

**Table 2:** Anthropometric data of **girls** in each age group of 6 European countries. (BE = Belgium; BG = Bulgaria; FI = Finland; GR = Greece; HU = Hungary; ES = Spain).

Prevalence					
GIRLS	Age-Group [year]	*n*	Weight [kg]	Height [cm]	BMI [kg/m^2^]
**BE**	**6–6.99**	**129**	23.23 [22.70–23.76]	121.03 [120.09–121.97]	15.81 [15.57–16.04]
	**7–7.99**	**374**	25.42 [25.02–25.82]	125.83 [125.26–126.40]	16.00 [15.82–16.18]
	**8–8.99**	**606**	28.99 [28.57–29.41]	132.06 [131.59–132.54]	16.54 [16.36–16.72]
	**9–9.99**	**580**	32.43 [31.88–32.98]	137.21 [136.70–137.72]	17.13 [16.91–17.35]
**BG**	**6–6.99**	**41**	25.65 [24.30–26.99]	122.10 [120.51–123.70]	17.15 [16.38–17.92]
	**7–7.99**	**382**	27.17 [26.62–27.72]	126.86 [126.31–127.42]	16.78 [16.52–17.04]
	**8–8.99**	**721**	30.79 [30.33–31.25]	132.42 [132.01–132.84]	17.46 [17.25–17.66]
	**9–9.99**	**783**	34.46 [33.94–34.99]	137.77 [137.34–138.20]	18.06 [17.84–18.27]
**FI**	**6–6.99**	**8**	27.16 [23.41–30.92]	125.66 [124.33–127.00]	17.18 [14.93–19.42]
	**7–7.99**	**204**	27.70 [26.95–28.46]	127.59 [126.85–128.33]	16.87 [16.53–17.22]
	**8–8.99**	**423**	30.17 [29.60–30.74]	132.33 [131.77–132.90]	17.14 [16.89–17.39]
	**9–9.99**	**612**	33.61 [33.07–34.16]	137.97 [137.47–138.46]	17.56 [17.34–17.78]
**GR**	**6–6.99**	**232**	25.23 [24.57–25.89]	121.41 [120.72–122.09]	17.01 [16.68–17.34]
	**7–7.99**	**593**	28.24 [27.77–28.72]	126.65 [126.19–127.10]	17.48 [17.26–17.70]
	**8–8.99**	**931**	31.76 [31.33–32.20]	132.12 [131.74–132.51]	18.07 [17.88–18.26]
	**9–9.99**	**783**	35.34 [34.80–35.88]	137.45 [137.01–137.90]	18.57 [18.36–18.79]
**HU**	**6–6.99**	**34**	25.68 [23.97–27.39]	124.33 [122.30–126.36]	16.51 [15.74–17.28]
	**7–7.99**	**274**	27.01 [26.15–27.88]	125.86 [125.07–126.65]	16.88 [16.47–17.28]
	**8–8.99**	**526**	30.70 [29.98–31.41]	131.33 [130.73–131.92]	17.60 [17.30–17.90]
	**9–9.99**	**778**	34.52 [33.83–35.22]	137.42 [136.89–137.94]	18.05 [17.78–18.32]
**ES**	**6–6.99**	**160**	24.59 [23.92–25.25]	121.20 [120.43–121.98]	16.65 [16.33–16.98]
	**7–7.99**	**385**	27.20 [26.65–27.76]	126.11 [125.55–126.66]	16.99 [16.74–17.24]
	**8–8.99**	**603**	30.63 [30.10–31.16]	131.72 [131.25–132.19]	17.52 [17.30–17.74]
	**9–9.99**	**521**	34.02 [33.33–34.70]	136.85 [136.31–137.40]	18.01 [17.74–18.28]

The means of weight-, height- and BMI values of girls separately in 4 age categories during the project period.

**Figure 4:**
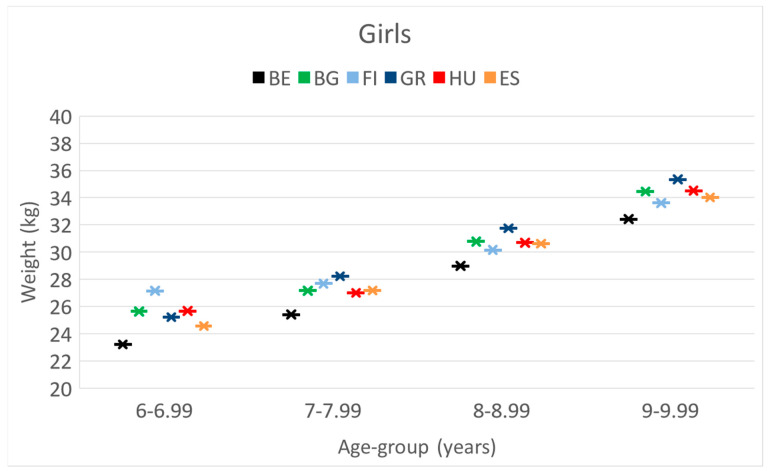
The increase in the mean weight [kg] of girls by their age [years] in the 6 countries. (BE = Belgium; BG = Bulgaria; FI = Finland; GR = Greece; HU = Hungary; ES = Spain).

**Figure 5:**
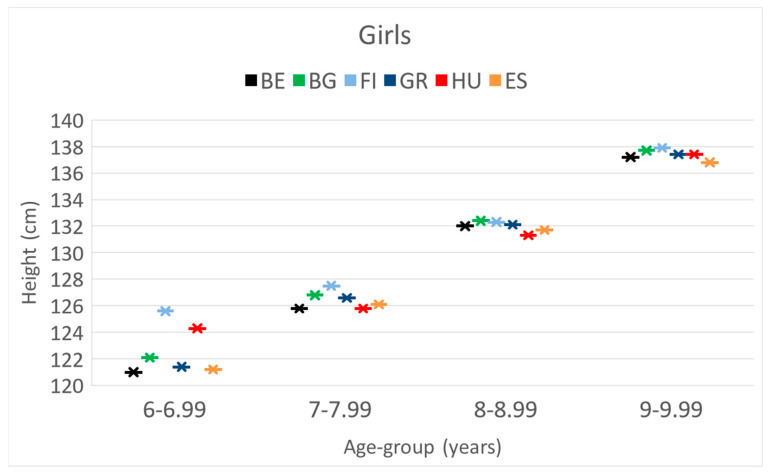
The increase in the mean height [cm] of girls by their age [years] in the 6 countries. (BE = Belgium; BG = Bulgaria; FI = Finland; GR = Greece; HU = Hungary; ES = Spain).

**Figure 6:**
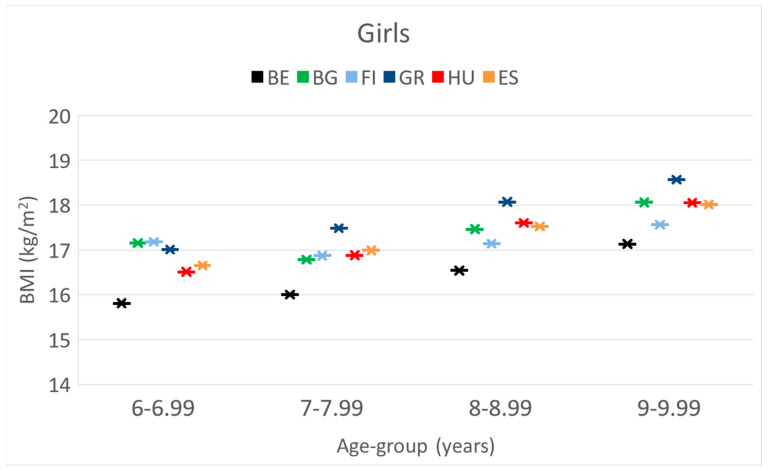
The changes in the mean BMI [kg/m^2^] by age [years] of girls in the 6 countries. (BE = Belgium; BG = Bulgaria; FI = Finland; GR = Greece; HU = Hungary; ES= Spain).

These differences and trends are more visible in Figure 4, Figure 5 and Figure 6 as well.

The distribution of overweight and obese children was different in the six countries examined. Greek boys represented the greatest proportion among overweight boys for all age cohorts (45–53%), followed by the Hungarians (34–38%), Spanish boys (36–37%) were the third, Bulgarians (30–36%) were the fourth, Finnish had the fifth position (31–34%), while Belgian boys (17–19%) had the lowest proportion above percentiles of 85.

**Figure 7:**
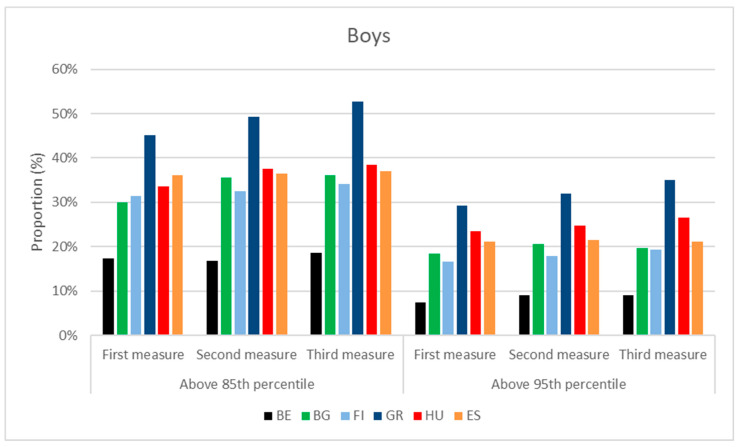
BMI distribution of boys at the time of 3 consecutive anthropometric measurements, performed during the project. (BE = Belgium; BG = Bulgaria; FI = Finland; GR = Greece; HU = Hungary; ES = Spain).

The distributions of obese boys above the 95 percentile were the same. The ranking list was led by the Greeks (29–35%), followed by the Hungarians (25–27%), thereafter, the Spanish (21%) and Bulgarian (18–20%). The last two groups were the Finnish (17–19%) and the Belgian boys (7–9%). The graphs are presented in Figure 7.

Among the population of girls, the Greeks represented the greatest proportion in the overweight range, depending on the time of measurements; in total, 41–44% of them were above the percentile of 85. The Spanish were in the second position (32–34%), followed by the Bulgarian (31–33%) and Hungarian girls (29–31%), and thereafter Finnish (27–28%) and Belgian girls (20–21%).

**Figure 8:**
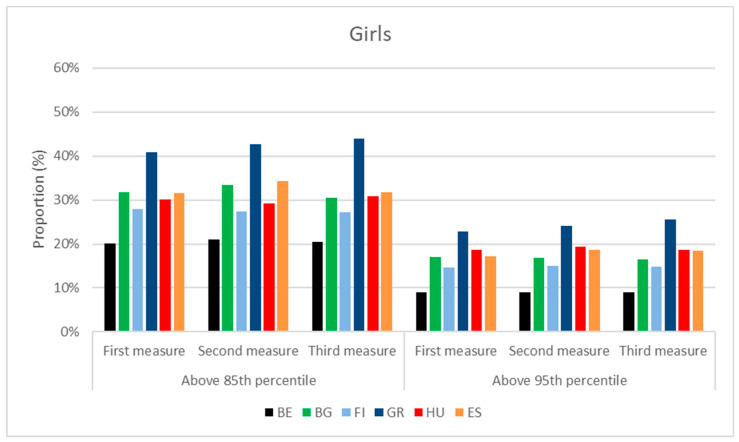
BMI distribution of girls at the time of 3 consecutive anthropometric measurements, performed during the project. (BE = Belgium; BG = Bulgaria; FI = Finland; GR = Greece; HU = Hungary; ES = Spain).

The trend in the distributions of obese girls above the 95 percentile was similar. Greek girls (23–25%) were followed by the Hungarians (19%), Spanish (17–18%) and Bulgarians (16–17%). Fifteen percent of Finnish and 9% of Belgian girls were considered obese. Their distributions are presented in Figure 8.

## 5. Discussion

### 5.1. Main Findings

The presented figures provide recent and professionally measured, reliable anthropometric data of a large populations of children from six European countries.

Some of them can be emphasized: Belgian boys had the lowest body weight and height, Greek boys had the highest body weight, and Finnish had the highest body height in almost all age cohorts. The highest proportion of overweight and obese boys was in Greece, followed by Hungarian, Spanish, Bulgarian and Finnish boys. Belgian boys had the lowest ratio in both categories.

Among girls, the Greeks had the highest, the Belgians had the lowest body weight, and the Finnish were the highest in all age categories. The ratio in the overweight range was the highest in Greece, followed by Spanish, Bulgarian and Hungarian girls, who were second in the obese category. Finnish girls had lower and Belgian girls had the lowest ratio in both BMI categories.

These small differences found between countries could be explained by the genetic background and environmental differences between these nations, similar to the anthropometric data of their adult populations [17]. Within these six countries, native populations are dominant, and a higher ratio of immigrants is present only in Belgium and Spain without unknown genetic influences.

### 5.2. Comparison to Previous Research

There have been many studies with anthropometric measurements of children. Almost all similar anthropometric data were recorded from eight different European countries by the *IDEFIC Study* [18]. The *WHO Child Growth Standards* described data between 5- and 19-year aged children [19]. Most of our data correspond to these and to the Finnish survey [20]. None of these studies compared national data, while only a small overlap was observed between the countries involved. Most of them had much fewer participants.

Some of the follow-up projects have proved the importance of school environment and individual lifestyle on children. In the COSI studies (*European Childhood Obesity Surveillance Initiative*), higher scores corresponded to higher support for a healthy school nutrition environment. Bulgaria, Greece and Hungary were among the low-score countries [21].

Anthropometric data recorded in the consecutive COSI studies (children aged 7–9 years) showed differences between countries. The order of overweight boys was: Greece and Spain (43%), Bulgaria (30%), Finland and Hungary (28%). These figures for obesity were from Greece (20%), Spain (18%), Bulgaria (16%), Hungary (14%) and Finland (12%). Among the pupils of the other gender, 41% of the Spanish, 37% of Greeks, and 28% of the Bulgarian and Hungarian girls were overweight, while only 26% were found in Finland. The measured ratio of obesity among girls was: 16% in Spain, 14% in Greece, 12% in Bulgaria and Hungary, and only 8% in Finland. In a very small ratio, obesity was less frequent among children of higher-educated parents [22]. Compared to our findings, the leading countries were the same; differences could be considered for the different cohorts examined. Considering the normal growth of children, differences in body parameters between genders were smaller than in the later age among adolescents [23].

The EU *Childhood Obesity Project* (CHOP) had a 5 year long follow-up period. Its main finding was that higher physical activity and lower sedentary behavior were the most effective tools for obesity prevention [24]. Different methods of recruitment could result in similar findings as well [25,26]. The methodology of these studies differed from the *Feel4Diabetes Study*, although there was wide agreement on the appropriate tools for obesity prevention and the importance of the topic.

### 5.3. Strengths and Limitations of the Study

The strengths of our study included a large sample size of measured children and a standardized method of measurement (conducted by trained researchers, not based on parents’ reports). We did not find data sources from other countries where the real representation of national cohorts would be clearly described to help the appropriate comparison. To the best of our knowledge, no studies have been previously conducted investigating and following parallel the anthropometric parameters of children in more European countries.

This presentation of data had many limitations:

There were differences in the number of children measured at different age groups in different countries. In some of them, certain age groups were underrepresented.

Due to the nature of the collected and presented data, comparisons between different strata should be handled with special caution because, when several comparisons were made, the probability of the family-wise error could increase, which could lead to the production of false discoveries, therefore distorting the understanding of the results.

The populations examined could not be considered representative at a national level, although no comparable national data were found even without representatives. Although in some countries, low SES areas were targeted, the educational level of parents and their economic circumstances were not surely homogeneous. The socio-economic circumstances of the children’s families may influence their individual somatic development.

While the *F4D study* had only a 2-year-long follow-up period, the data of these children was not available before and after the project.

The ongoing F4D project may have a certain effect on the parameters of children.

The year-long age cohorts compared (i.e., 6.0–6.99 y, etc.) could be too wide in this growing period of children, but we had no other option for the categorization of these measurements.

Mainly comparisons between countries and trends were described. It would be irrational to count the statistical analysis between countries; therefore, only some Hungary-related findings were described.

## 6. Conclusions

The main findings of this study support the importance of early obesity prevention. While other published outcomes of this 2-year long project are not presented here, it became clear that life-long interventions for the entire population and more political-economical support are needed, in addition to appropriate education on healthy lifestyles in schools, within the family and in the mass media as well.

## Data Availability

The datasets generated and/or analyzed during the current study are not publicly available due to the contract between the partners and the funding body but are available from the corresponding author on reasonable request.

## References

[1] Bleich S.N., Segal J., Wilson R., Wang J. (2013). Systematic Review of Community-Based Childhood Obesity Prevention Studies. Pediatrics.

[2] Pandita A., Sharma D., Pandita D., Pawar S., Tariq M., Kaul A. (2016). Childhood obesity: Prevention is better than cure. Diabetes Metab. Syndr. Obes. Targets Ther..

[3] Koletzko B., Fishbein M., Lee W.S., Moreno L., Mouane N., Mouzaki M., Verduci E. (2020). Prevention of Childhood Obesity: A Position Paper of the Global Federation of International Societies of Paediatric Gastroenterology, Hepatology and Nutrition (FISPGHAN). JPGN.

[4] Antwi F., Fazylova N., Garcon M.C., Lopez L., Rubiano R., Slyer J.T. (2012). The effectiveness of web-based programs on the reduction of childhood obesity in school-aged children: A systematic review. JBI Libr. Syst. Rev..

[5] Weihrauch-Blüher S., Kromeyer-Hauschild K., Graf C., Widhalm K., Korsten-Reckf U., Jödickeg B., Markerta J., Müller M.J., Moss A., Wabitschj M. (2018). Current Guidelines for Obesity Prevention in Childhood and Adolescence. Obes. Facts.

[6] Anthropometric Measurements–Guideline 4. California Department of Health Care Services, Systems of Care Division Child Health and Disability Prevention Program, Health Assessment Guidelines March 2016. https://www.dhcs.ca.gov/services/chdp/Documents/HAG/4AnthropometricMeasure.pdf.

[7] Wicklow B.A., Becker A., Chateau D., Palmer K., Kozyrskij A., Sellers E.A.C. (2015). Comparison of anthropometric measurements in children to predict metabolic syndrome in adolescence: Analysis of prospective cohort data. Int. J. Obes..

[8] Growth Reference Data for 5–19 Years, Indicators, BMI for Age (5–19 Years). https://www.who.int/tools/growth-reference-data-for-5to19-years/indicators/bmi-for-age.

[9] Children’s Nutrition Research Center, Children’s BMI Percentile for Age Calculator. https://www.bcm.edu/cnrc-apps/bodycomp/bmiz2.html.

[10] Body Composition Laboratory Age Based Pediatric Growth Reference Charts. https://www.bcm.edu/bodycomplab/Flashapps/bmiVAgeChartpage.html.

[11] Pretorius S.S., Neophytou N., Watson E.D. (2019). Anthropometric profiles of 8–11 years old children from a low-income setting in South Africa. BMC Public Health.

[12] Cole T.J., Bellizzi M.C., Flegal K.M., Dietz W.H. (2000). Establishing a standard definition for child overweight and obesity worldwide: International survey. BMJ.

[13] Feel4Diabetes Study. https://feel4diabetes-study.eu/.

[14] Manios Y., Lambrinou C.P., Mavrogianni C., Cardon G., Lindström J., Iotova V., Tankova T., Rurik I., Stappen V.V., Kivelä J. (2020). Lifestyle Changes Observed among Adults Participating in a Family- and Community-Based Intervention for Diabetes Prevention in Europe: The 1(st) Year Results of the Feel4Diabetes-Study. Nutrients.

[15] Manios Y., Androutsos O., Lambrinou C.P., Cardon G., Lindstrom J., Annemans L., Mateo-Gallego R., de Sabata M.S., Iotova V., Kivela J. (2018). A school- and community-based intervention to promote healthy lifestyle and prevent type 2 diabetes in vulnerable families across Europe: Design and implementation of the Feel4Diabetes-study. Public Health Nutr..

[16] Liatis S. (2020). Feel4Diabetes-study. Methodological procedures followed in a school-and community-based intervention to prevent type 2 diabetes in vulnerable families across Europe: The Feel4Diabetes-study. BMC Endocr. Disord..

[17] World Health Organization (WHO) Developments in Nutrition, Physical Activity and Obesity in the WHO European Region. https://www.euro.who.int/en/health-topics/disease-prevention/nutrition/country-work.

[18] Hunsberger M., Mehlig K., Börnhorst C., Hebestreit A., Moreno L., Veidebaum T., Kourides Y., Siani A., Molnár D., Sioen I. (2015). Dietary Carbohydrate and Nocturnal Sleep Duration in Relation to Children’s BMI: Findings from the IDEFICS Study in Eight European Countries. Nutrients.

[19] de Onis M., Onyango A.W., Borghi E., Siyam A., Nishida C., Siekmann J. (2007). Development of a WHO growth reference for school-aged children and adolescents. Bull. World Health Organ..

[20] Finnish Institute for Health and Welfare, FinChildren Register Monitoring. Monitoring Child, Adolescent and Family Health and Well-Being. http://www.terveytemme.fi/finlapset/en/index.html.

[21] Wijnhoven T.M.A., van Raaij J.M.A., Sjöberg A., Eldin N., Yngve A., Kunešová M., Starc G., Rito A.I., Duleva V., Hassapidou M. (2014). WHO European Childhood Obesity Surveillance Initiative: School Nutrition Environment and Body Mass Index in Primary Schools. Int. J. Environ. Res. Public Health.

[22] (2012). WHO European Childhood Obesity Surveillance Initiative (COSI) Report on the Fourth Round of Data Collection, 2015–2017; World Health Organization: Geneva, Switzerland. https://apps.who.int/iris/bitstream/handle/10665/341189/WHO-EURO-2021-2495-42251-58349-eng.pdf.

[23] Vehrs P.R., Fellingham G.W., McAferty A., Kelsey L. (2022). Trends in BMI Percentile and Body Fat Percentage in Children 12 to 17 Years of Age. Children.

[24] Schwarzfischer P., Gruszfeld D., Socha P., Luque V., Closa-Monasterolo R., Rousseaux D., Moretti M., Mariani B., Verduci E., Koletzko B. (2018). Longitudinal analysis of physical activity, sedentary behaviour and anthropometric measures from ages 6 to 11 years. Int. J. Behavioral. Nutr. Phys. Act..

[25] Tobisch B., Blatniczky L., Schusterova I., Kovács L., Barkai L. (2021). Insulin resistance and its effects in children and adolescents. Orv. Hetil..

[26] Rose J., Lynn K., Akister J., Maxton F., Redsell S.A. (2021). Community midwives’ and health visitors’ experiences of research recruitment: A qualitative exploration using the Theoretical Domains Framework. Prim. Health Care Res. Dev..

